# Efficacy and Mechanism of Action of Marine Alkaloid 3,10-Dibromofascaplysin in Drug-Resistant Prostate Cancer Cells

**DOI:** 10.3390/md18120609

**Published:** 2020-12-01

**Authors:** Sergey A. Dyshlovoy, Moritz Kaune, Jessica Hauschild, Malte Kriegs, Konstantin Hoffer, Tobias Busenbender, Polina A. Smirnova, Maxim E. Zhidkov, Ekaterina V. Poverennaya, Su Jung Oh-Hohenhorst, Pavel V. Spirin, Vladimir S. Prassolov, Derya Tilki, Carsten Bokemeyer, Markus Graefen, Gunhild von Amsberg

**Affiliations:** 1 Laboratory of Experimental Oncology, Department of Oncology, Hematology and Bone Marrow Transplantation with Section Pneumology, Hubertus Wald-Tumorzentrum, University Medical Center Hamburg-Eppendorf, Martinistrasse 52, 20251 Hamburg, Germany; moritz.kaune@stud.uke.uni-hamburg.de (M.K.); j.hauschild@uke.de (J.H.); tobias.busenbender@gmx.de (T.B.); c.bokemeyer@uke.de (C.B.); g.von-amsberg@uke.de (G.v.A.); 2 Laboratory of Pharmacology, A.V. Zhirmunsky National Scientific Center of Marine Biology, Far Eastern Branch, Russian Academy of Sciences, Palchevskogo str. 17, 690041 Vladivostok, Russian; 3 Martini-Klinik, Prostate Cancer Center, University Hospital Hamburg-Eppendorf, Martinistrasse 52, 20251 Hamburg, Germany; s.oh-hohenhorst@uke.de (S.J.O.-H.); d.tilki@uke.de (D.T.); graefen@martini-klinik.de (M.G.); 4 School of Natural Sciences, Far Eastern Federal University, FEFU Campus, Ajax Bay 10, Russky Island, 690922 Vladivostok, Russian; pollianna_95@mail.ru (P.A.S.); zhidkov.me@dvfu.ru (M.E.Z.); 5 Department of Radiotherapy & Radiation Oncology, Hubertus Wald Tumorzentrum–University Cancer Center Hamburg (UCCH), University Medical Center Hamburg-Eppendorf, Martinistrasse 52, 20251 Hamburg, Germany; m.kriegs@uke.de (M.K.); k.hoffer@uke.de (K.H.); 6 UCCH Kinomics Core Facility, Hubertus Wald Tumorzentrum–University Cancer Center Hamburg (UCCH), University Medical Center Hamburg-Eppendorf, Martinistrasse 52, 20251 Hamburg, Germany; 7 Laboratory of Proteoform Interactomics, Institute of Biomedical Chemistry, Pogodinskaya str. 10/8, 119121 Moscow, Russian; k.poverennaya@gmail.com; 8 Institute of Anatomy and Experimental Morphology, University Medical Center Hamburg-Eppendorf, Martinistrasse 52, 20246 Hamburg, Germany; 9 Engelhardt Institute of Molecular Biology, Russian Academy of Sciences, Vavilova 32, 119991 Moscow, Russian; spirin.pvl@gmail.com (P.V.S.); prassolov45@mail.ru (V.S.P.); 10 Department of Urology, University Hospital Hamburg-Eppendorf, Martinistrasse 52, 20251 Hamburg, Germany

**Keywords:** fascaplysin, prostate cancer, JNK1/2, natural products, synergism

## Abstract

Efficacy and mechanism of action of marine alkaloid 3,10-dibromofascaplysin (DBF) were investigated in human prostate cancer (PCa) cells harboring different levels of drug resistance. Anticancer activity was observed across all cell lines examined without signs of cross-resistance to androgen receptor targeting agents (ARTA) or taxane based chemotherapy. Kinome analysis followed by functional investigation identified JNK1/2 to be one of the molecular targets of DBF in 22Rv1 cells. In contrast, no activation of p38 and ERK1/2 MAPKs was observed. Inhibition of the drug-induced JNK1/2 activation or of the basal p38 activity resulted in increased cytotoxicity of DBF, whereas an active ERK1/2 was identified to be important for anticancer activity of the alkaloid. Synergistic effects of DBF were observed in combination with PARP-inhibitor olaparib most likely due to the induction of ROS production by the marine alkaloid. In addition, DBF intensified effects of platinum-based drugs cisplatin and carboplatin, and taxane derivatives docetaxel and cabazitaxel. Finally, DBF inhibited AR-signaling and resensitized AR-V7-positive 22Rv1 prostate cancer cells to enzalutamide, presumably due to AR-V7 down-regulation. These findings propose DBF to be a promising novel drug candidate for the treatment of human PCa regardless of resistance to standard therapy.

## 1. Introduction

In the past decades, treatment of metastatic prostate cancer (mPCa) tremendously improved resulting in increased overall survival (OS) and better quality of life of the patients. However, despite the high efficacy of androgen receptor targeting agents (ARTA) and docetaxel in the hormone naïve stage of disease, loss of efficacy of standard therapies is eventually observed in the course of treatment reflected by reduced PSA-responses and a decreased OS with each additional treatment line [1,2,3]. To date, different mechanisms of resistance have been identified. Thus, modification of the androgen receptor (AR) including amplification, mutation, and alternative splice variants as well as PTEN loss were found to be associated with decreased sensitivity to ARTAs, while up-regulation of p-glycoprotein and the induction of life-prolonging autophagy mediate resistance to standard chemotherapy [2]. To date, treatment strategies to overcome drug resistance in mPCa are limited and novel agents effectively targeting drug-resistant prostate cancer are urgently needed.

Marine invertebrates are a rich source of new molecules having a unique structure and promising biological activity [4,5]. A significant proportion of these compounds are cytotoxic to cancer cells making them promising candidates for further development. By the end of 2020, there will be 10 clinically-approved anticancer drugs created on the bases of small molecules isolated from marine organisms [6,7]. In addition, many more are in different stages of clinical and preclinical development and around 1000 new structures are reported every year [8,9].

Fascaplysin is a red bioactive pigment initially isolated from the marine sponge *Fascaplysinopsis* sp. [10]. This alkaloid possesses a 12*H*-pyrido[1,2-*a*:3,4-*b’*]diindole core [11] and reveals a broad spectrum of biological activities, including antifungal, antibacterial, antiviral, antimalarial, and antitumor effects [11,12,13]. Its antitumor activity was evaluated in different human cancer cells lines including lung, prostate, breast, and colorectal cancer, melanoma and leukemia [13,14,15,16,17,18,19,20]. Fascaplysin and similar alkaloids seem to mediate their activity by inhibition of cyclin-dependent kinase 4 (CDK4) and intercalation with dsDNA, which leads to a G1-phase cell cycle arrest and ultimately to the apoptotic cancer cell death [11,12,21,22]. However, recently it was reported that fascaplysin can kill cancer cells independently from the presence and activity of CDK4, suggesting other molecular targets to be involved in its mechanism of action [17]. Indeed, several other molecular targets and cellular processes, both pro-apoptotic and pro-survival, have also been reported to be affected by this marine alkaloid. Thus, fascaplysin was capable to inhibit VEGFR2, TRKA, surviving, and HIF-1α [17]. In lung and colorectal cancer cells, synergistic effects with AKT and AMPK inhibitors were observed, emphasizing its possible implication in combinational therapy [23]. Additionally, an induction of cytoprotective autophagy was found in human breast cancer cells as well as human vascular endothelial cells treated with fascaplysin and its derivatives [18,19]. This event was linked to the PI3K/AKT/mTOR signaling inhibition in the treated cells [18,24]. Autophagy is often induced in cancer cells exposed to chemotherapeutics as one of the basic survival mechanisms, helping the cells to overcome stress conditions [25,26].

Several halogenated fascaplysin derivatives were found to exhibit antitumor activity as well. Thus, 3-chlorofascaplysin suppressed angiogenesis and tumor growth of breast cancer cells [18]. The brominated derivatives, i.e., 3- and 10-bromofascaplysins induced a caspase-dependent apoptosis in human leukemia cells at nanomolar concentrations [13]. Additionally, these compounds were active in rat C6 glioma cells model [27]. Of note, in this experiment the brominated derivatives were more active in comparison with “mother” fascaplysin molecule [27].

3,10-Dibromofascaplysin (DBF) is a novel halogenated fascaplysin alkaloid initially isolated from the marine sponge *Fascaplysinopsis reticulata* [28] and later synthesized by our group [20]. Recently, we identified DBF to be active in human prostate cancer cells during a small-scale screening of semi-synthetic fascaplysin derivatives. In contrast to the other synthesized derivatives, DBF revealed a smooth cytotoxicity profile, suggesting a wide therapeutic window [20]. In addition, DBF was found to affect cellular metabolism, which further leads to cancer cell death [20]. In the current research we evaluated the activity of DBF in human prostate cancer cell lines harboring different levels of drug resistance to currently available standard therapies. Mechanism of action and molecular targets were examined by a kinome profiling approach.

## 2. Results and Discussion

### 2.1. 3,10-Dibromofascaplysin (DBF) Induces Apoptotic Cell Death of Drug-Resistant Prostate Cancer Cells

**Figure 1:**
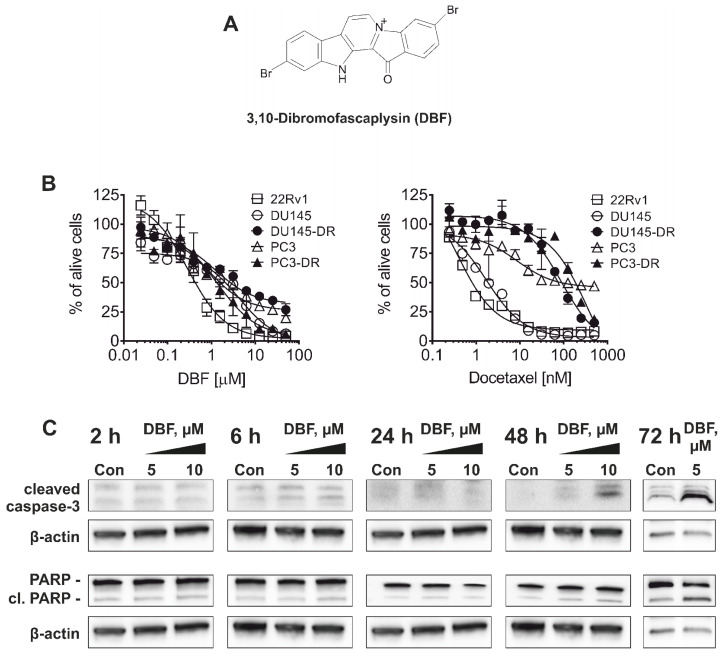
**Cytotoxicity and selectivity of DBF**. (**A**), The structure of DBF. (**B**), Cytotoxicity profiles of DBF in human prostate cancer cell lines resistant to hormone therapy or docetaxel. Cell viability was measured using MTT assay following 72 h of incubation. (**C**), Western blotting analysis of the protein expression in 22Rv1 cells treated with DBF for indicated time. β-actin was used as a loading control.

Overcoming drug resistance is a major challenge in the treatment of advanced prostate cancer. 3,10-Dibromofascaplysin (DBF, Figure 1A)—a new halogenated fascaplysin—showed promising activity in previous screening experiments [20]. Therefore, we evaluated cytotoxicity of this marine alkaloid in different human drug-resistant prostate cancer cell lines in vitro.

22Rv1, PC3 and DU145 cells reveal resistance to AR-targeting therapies e.g., abiraterone and enzalutamide. In 22Rv1 cells, resistance is mediated by the expression of AR splice variant 7 (AR-V7) [29], which lacks an androgen binding site and induces permanent auto-activation of the ARs [30]. PC3 and DU145 cells lack AR expression and thus do not require androgens for growth and proliferation [29]. DBF was found to be cytotoxic in all cell lines investigated at micro- and nanomolar concentrations with the highest activity in 22Rv1 cells (Table 1). The docetaxel-resistant PC3 and DU145 sublines (PC3-DR and DU145-DR) were generated using continuous incubation of PC3 and DU145 with increasing concentrations of docetaxel until reaching a concentration of 12.5 nM as previously described [31]. Notably, the PC3-DR and DU145-DR cells are ~50-fold less sensitive to docetaxel compared to their parental cell lines (Figure 1B). Remarkably, IC_50_ of DBF in PC3-DR cells was only 2-fold higher compared to PC3 cells, and DU145-DR cells were even more sensitive to DBF than DU145 cells suggesting no cross-resistance between docetaxel and DBF (Figure 1B, Table 1).

In a next step, we evaluated the mechanism of action of DBF in prostate cancer. 22Rv1 cells were chosen as they revealed the highest sensitivity to the marine compound. First, apoptotic markers including poly(ADP-ribose)polymerase (PARP) and caspase-3 were examined to determine the character of cell death mediated by DBF (Figure 1C). In fact, cleavage of both proteins first appeared 48 h after treatment of 22Rv1 cells at concentrations of 5 and 10 µM. Hence, these conditions were chosen for further experiments.

### 2.2. DBF Induces Alterations of Protein Tyrosine Kinases Activity

**Figure 2:**
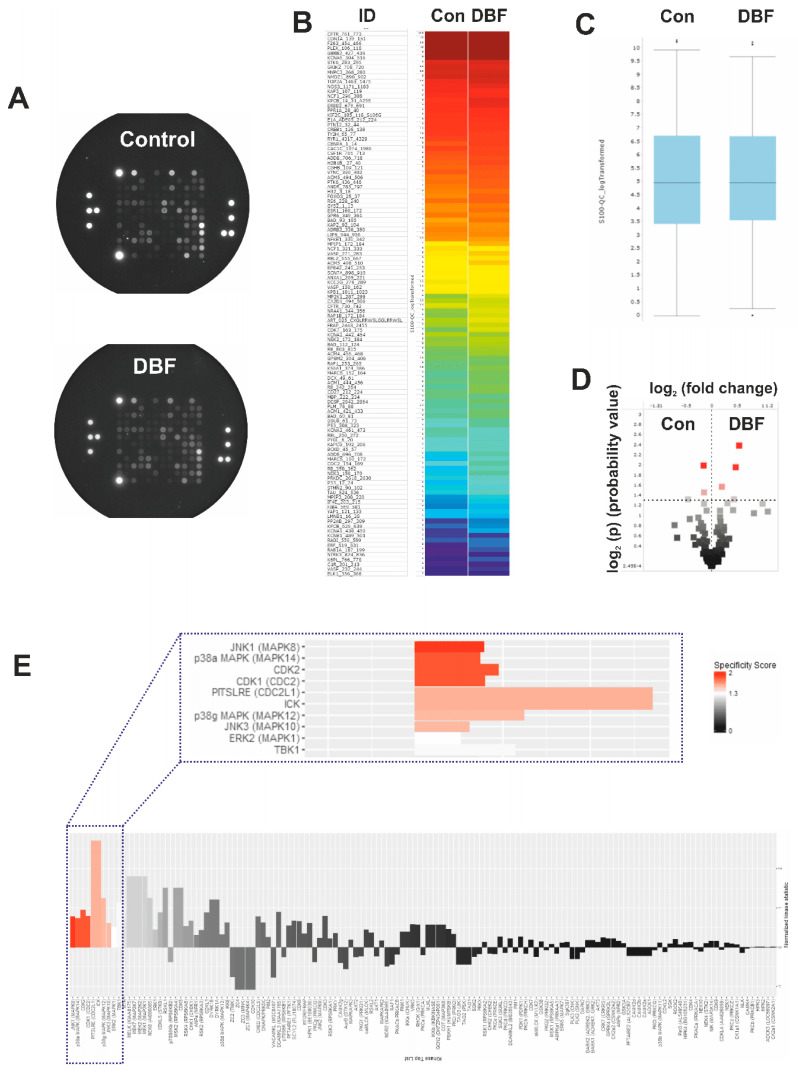
**Functional kinome profiling of serine/threonine kinases (STKs)**. 22Rv1 cells were treated with DBF for 2 h; proteins were extracted and analyzed using STK-PamChip^®^ (sequence-specific peptide phosphorylation assay) and anti-phospho-STK antibodies. (**A**), Microphotographs of STK-PamChip^®^. The generated data is represented as heat-map plot (**B**), box plots (**C**), or volcano-plot (**D**). Red dots indicate peptide substrates having significantly increased phosphorylation in comparison to control samples (log_2_(p) > 1.3, dotted line, (**D**)). (**E**), Upstream analysis of the treatment-affected kinases in 22Rv1 cells. Normalized kinase statistic > 0 indicates higher kinase activity in DBF-treated cells; specificity score > 1.3 indicates statistically significant changes.

Protein kinases are important target molecules for various anticancer drugs [32]. They catalyze the phosphorylation of target proteins thereby modulating their activity. Serine/threonine kinases (STK) belong to a group of protein kinases, involved in critical processes related to both, cellular death and survival [33]. In fact, modulation of STKs activity has been identified as a mechanism of action of a number of clinically-approved drugs, such as cobimetinib, palbociclib, axitinib, sunitinib, and others [33]. Hence, we assessed the effect of DBF on STKs activity by functional kinomics assay using the PamTechnology^®^ (http://www.pamgene.com, Figure 2A–E) [34]. This method allows to determine specific STK activity changes in living cells. Short-term treatment for 2 h was chosen in order to minimize the detection of unspecific effects secondary to cell death related events. Results are shown for the control group vs. the treated group, as a log2 of signal intensity per peptide (Figure 2A–D). No significant changes of the overall STKs activity were observed between the groups (Figure 2C). However, further analyses of specific STK potentially affected by DBF predicted an activation of JNK1 kinase (Figure 2E), belonging to the group of the mitogen activated protein kinases (MAPKs). Additionally, other MAPKs, such as p38 and ERK1, were also predicted to be activated by DBF, however, with a lower specificity score (Figure 2E). The above mentioned MAPKs are associated with different cancer-related processes, however, with ambiguous impact on cancer cell elimination and growth inhibition [32,35]. In prostate cancer, MAPKs were reported to have an important impact on tumor growth [35,36,37]. Additionally, a crosstalk with AR-signaling has been demonstrated, especially for JNK1/2 [38,39,40]. Thus, the changes of JNK1/2 activity and other MAPKs were further examined.

### 2.3. Validation of Kinome Analysis Data

**Figure 3:**
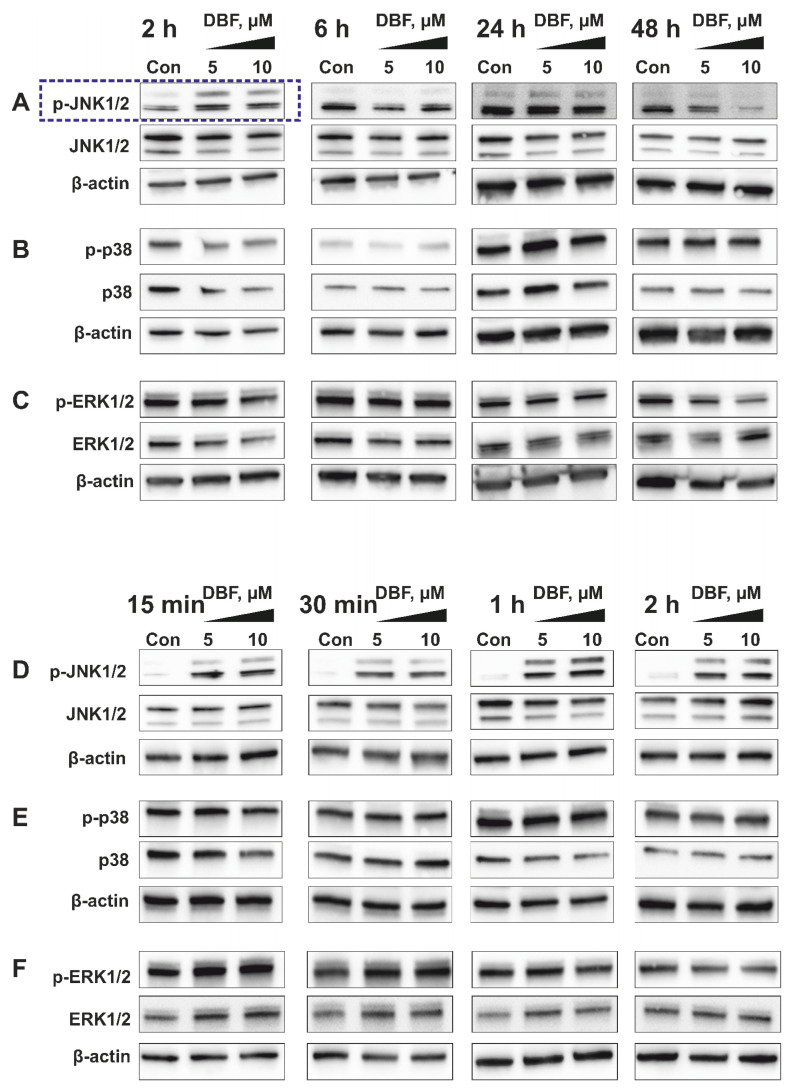
**Validation of kinome analysis data using Western blotting**. Western blotting analyses of the phosphorylated and total JNK1/2 (**A**,**D**), p38 (**B**,**E**), and ERK1/2 (**C**,**F**) kinases in 22Rv1 cells treated with DBF for 2–48 h (**A**–**C**) or 15 min–2 h (**D**–**F**). β-actin was used as a loading control.

JNK1 was the top-ranked kinase predicted to be activated by DBF in prostate cancer 22Rv1 cells (Figure 2E). Hence, time-dependent Western blotting-based analyses of JNK1/2 activation were performed for further validation (Figure 3A). Indeed, a phosphorylation of JNK1/2 was only observed in PCa cells exposed to short-term DBF treatment (2 h), whereas no activation, or even phospho-JNK1/2 degradation was found after 6 h to 48 h (Figure 3A). More detailed examinations revealed a pronounced JNK1/2 phosphorylation already after 15 min of DBF treatment. Thus, JNK1/2 phosphorylation is one of the very first cellular events following drug exposure (Figure 3D), long before first apoptotic signs appear (48 h, Figure 1C). Due to a potential crosstalk of different MAPKs, we examined the effect of DBF on p38 and ERK1/2 MAPKs, which were also predicted to be affected by kinomic analysis (Figure 3B,C,E,F). Of note, no significant alterations of p38 and ERK1/2 were observed at any time point ranging from 15 min to 48 h of treatment (Figure 3B,C,E,F).

### 2.4. Role of JNK1/2 and Other MAPKs in Cytotoxic Effect of DBF

**Figure 4:**
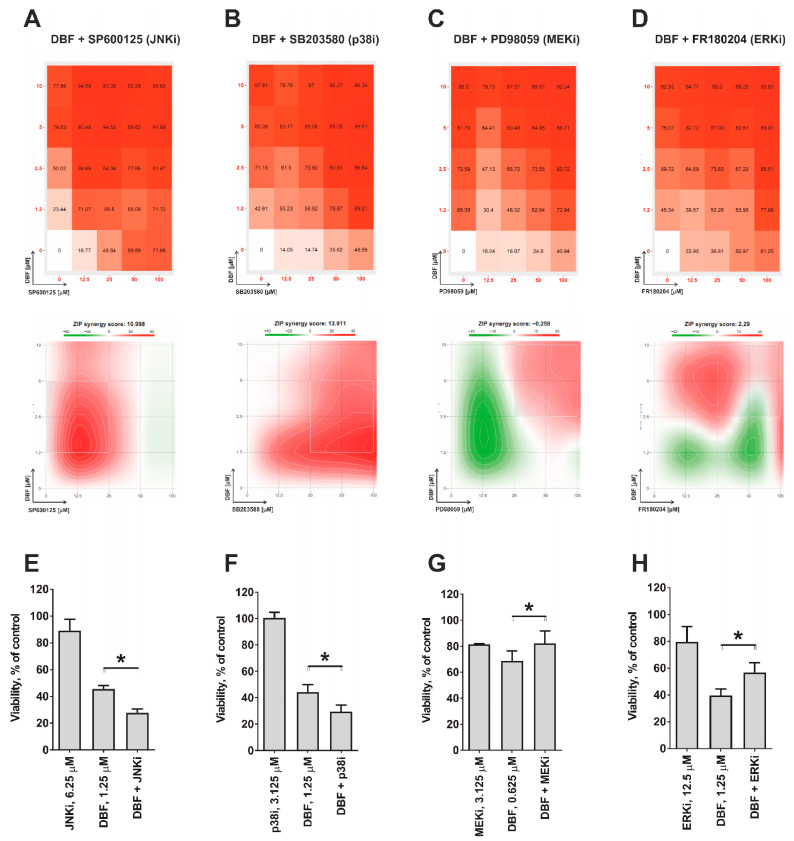
**Analysis of the effect on DBF in combination with MAPKi**. 22Rv1 cells were co-treated with DBF in combination with JNK1/2 inhibitor SP600125 (**A**,**E**), p38 inhibitor SB203580 (**B**,**F**), MEK1/2 inhibitor PD98059 (**C**,**G**), or ERK1/2 inhibitors FR180204 (**D**,**H**) for 48 h. The viability was measured using the MTT assay and the effect of the drug combination (synergism / additive effect / antagonism) was calculated and visualized using SynergyFinder 2.0 software and a ZIP reference model (**A**–**D**); or presented as of cell viability in % of control (**E**–**H**). Red regions indicate synergism; white—additive effect; green—antagonism (**A**–**D**). * *p* < 0.05, one-way ANOVA test.

In malignant conditions, the impact of JNK1/2, p38, and ERK1/2 MAPKs depend on the cellular context as well as the nature of stimuli ranging from pro-survival to pro-apoptotic effects [41]. In order to determine the role of JNK1/2 and the other MAPKs in execution of the biological effects of DBF, we applied a co-treatment with specific inhibitors. Therefore, selective JNK1/2 inhibitor SP600125 (Figure 4A), p38 inhibitor SB203580 (Figure 4B), MEK1 inhibitor PD98039 (Figure 4C), as well as ERK1/2 inhibitor FR180204 (Figure 4D) were tested. Note, MEK1/2 kinase exclusively and directly activates ERK1/2 [42] indicating that an inactivation of MEK1/2 leads to an inhibition ERK1/2 [42]. Due to the cytotoxic nature of most of the inhibitors mentioned above, we used combinations of several active concentrations to determine a synergistic or antagonistic effect of the inhibitors on cytotoxic activity of DBF. The data were generated using MTT assay and were further analyzed using SynergyFinder 2.0 software and a Zero interaction potency (ZIP) reference model [43] (Figure 4A–D). We generated heat-maps for the effects of DBF and the MAPK inhibitors alone, and for their respective combinations (Figure 4A–D). Our analyses revealed pronounced synergistic effects of DBF with JNKi (SP600125) and p38i (SB203580) suggesting a pro-survival role of both kinases in the cellular response to DBF treatment (Figure 4A,B). In contrast, the MEK/ERKi (PD98039/FR180204) antagonized cytotoxic effects of DBF (Figure 4C,D) indicating ERK1/2 to exert a cytotoxic function in DBF-treated 22Rv1 cells. Additionally, to confirm these data we applied the lower non-cytotoxic concentrations of the inhibitors in order to avoid unspecific cytotoxicity-related effects (Figure 4E–H). Thus, in line with the above described results (Figure 4A–D), co-treatment with JNKi and p38i increased the cytotoxic effects of DBF, while in contrast combination with MEKi and ERKi reduced DBF mediated cytotoxicity (Figure 4E–H).

Interestingly, in our experiments we observed a transient (temporal) activation of JNK1/2, which takes place within first two hours, and then decreases to the basal level by the time point of 6 h (Figure 3A,D). A number of previous studies report that activation of MAPKs may have either transient or sustained character in the same model, depending on stimulus nature (reviewed in [44]). Moreover, the time course of MAPK activation may be critical for the specific outcome of this event as well as for the cellular fate [44,45,46,47]. In particular, it has been shown that sustained activation of JNK normally leads to the apoptotic program activation, whereas temporal short-term activation of this MAPK stimulates the cellular survival [45]. In line with this, the observed short-term activation of JNK1/2 in DBF-stimulated cells (Figure 3A,D) which was identified as a pro-survival event (Figure 4A,E)

In summary, DBF treatment is accompanied by JNK1/2 activation. The inhibition of this process could synergistically increase an anticancer effect of the marine alkaloid. Although, no regulation of p38 or ERK1/2 was observed in our experiments, the generated results suggest an involvement of both kinases in the maintenance of cellular processes, which are important for survival and death of the drug-treated cancer cells, correspondingly (Figure 4C,D).

### 2.5. Effect of DBF in Combination with Platinum and Taxane Agents

**Figure 5:**
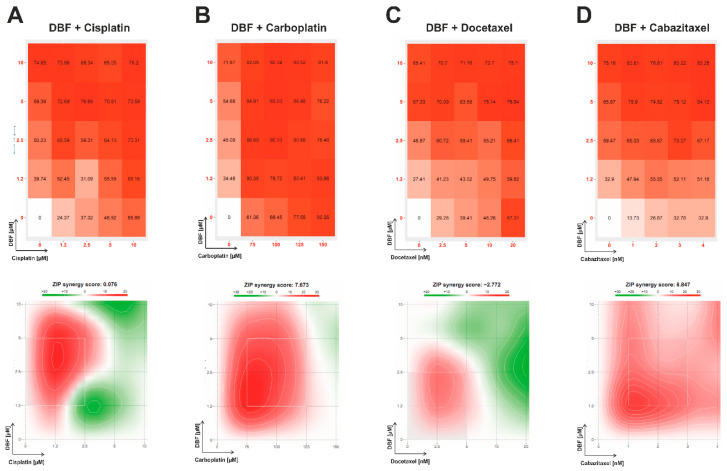
**Analysis of the effect on DBF in combination with platinum and taxane drugs**. 22Rv1 cells were co-treated with DBF in combination with cisplatin (**A**), carboplatin (**B**), docetaxel (**C**), or cabazitaxel (**D**) for 48 h. The viability was measured using the MTT assay and the effect of the drug combination (synergism/additive effect/antagonism) was calculated and visualized using SynergyFinder 2.0 software and a ZIP reference model. Red regions indicate synergism; white—additive effect; green—antagonism.

Taxanes are routinely applied in advanced prostate cancer, while platine-based therapy shows pronounced activity in patients harboring DNA repair defects or aggressive variants of the disease. In order to determine the clinical relevance of DBF in potential combinational therapies, co-treatment with cisplatin and carboplatin (platinum drugs, DNA-binding agents inducing cross-links) (Figure 5A,B), as well as docetaxel and cabazitaxel (taxane derivates, which mediate a microtubuline stabilization) was performed (Figure 5C,D). The activity of the drugs in combinations was analyzed by SynergyFinder 2.0 software using a ZIP reference model.

Remarkably, for DBF combinations with all afore mentioned drugs pronounced synergistic effects were detected (Figure 5A–D). Of note, synergism was observed for the whole range of carboplatin and cabazitaxel concentrations (Figure 5B,D), whereas for combinations with cisplatin and docetaxel synergistic effects were observed only for low doses of the drugs (Figure 5A,C).

### 2.6. Effect of DBF in Combination with Olaparib

**Figure 6:**
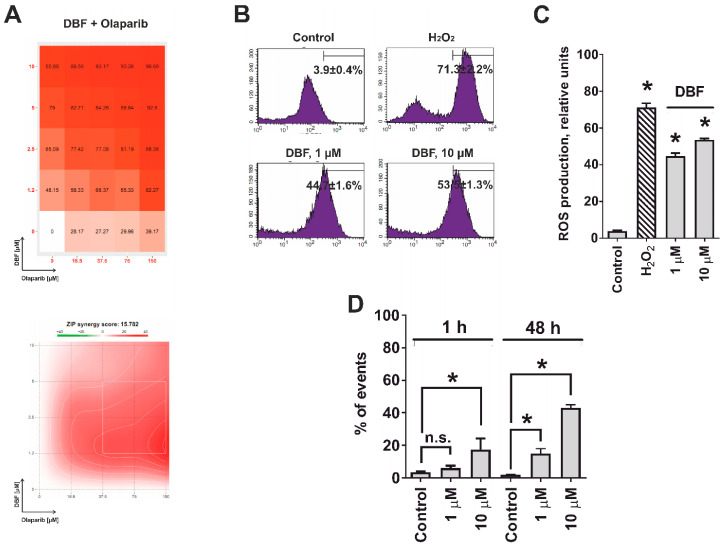
**Analysis of the effect on DBF on ROS production and DNA damage**. (**A**), 22Rv1 cells were co-treated with DBF in combination with olaparib for 48 h. The viability was measured using the MTT assay and the effect of the drug combination (synergism / additive effect / antagonism) was calculated and visualized using SynergyFinder 2.0 software and a ZIP reference model. Red regions indicate synergism; white—additive effect; green—antagonism. (**B**,**C**), Effect of DBF on ROS production in 22Rv1 cells following 1 h of treatment. The analysis was performed using CM-H_2_DCFDA staining and flow cytometry technique (**B**). The ROS level was quantified with Cell Quest Pro software (**C**). H_2_O_2_ (200 µM) was used as a positive control. (**D**), Analysis of DNA break in 22Rv1 cells treated with DBF for indicated time. n.s.—non-significant (*p* > 0.05), * *p* < 0.05, one-way ANOVA test.

We further examined the effects of DBF in combination with PARP inhibitor olaparib. Olaparib has recently been approved by the FDA for the treatment of patients with deleterious germline or somatic homologous recombination repair (HRR) gene-mutated metastatic castration-resistant PCs. PARP holds major functions on DNA repair of single strand breaks (ssDNA), induced by different stress factors [48]. Inhibition of ssDNA reparation by PARP inhibitors results in DNA double strand (dsDNA) breaks eventually leading to synthetic cell death in cells carrying HRR defects [49,50,51]. 22Rv1 cells are known to bear a BRCA2 defect, the most frequent HRR alteration in PCa, revealing high sensitivity to olaparib [52]. Remarkably, DBF exhibited a strong synergistic effect when combined with olaparib at the whole range of concentrations of both drugs (Figure 6A). We hypothesized that this may be due to ssDNA breaks following treatment with DBF, potentially mediated by increased production of reactive oxygen species (ROS) [53]. Hence, we have examined the induction of ROS in 22Rv1 cells treated with DBF. Notably, a significant increase of the ROS level was detected already after 1 h of treatment with 1 µM of DBF (Figure 6B,C), whereas the DNA damage could be detected only after longer time of treatment with the same concentration of the drug (Figure 6D). Thus, the ROS induction is a primary effect of the alkaloid in cancer cells. Moreover, the previously described JNK1/2 activation (Figure 2A,B), may also result from oxidative stress [54].

### 2.7. Effect of DBF on AR Signaling

**Figure 7:**
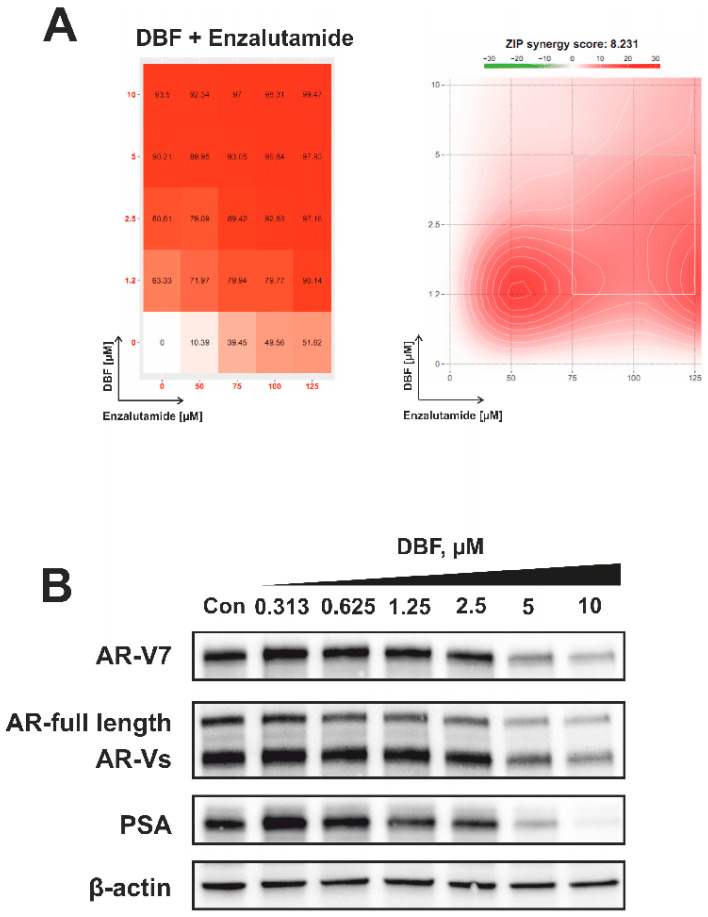
**Analysis of the effect on DBF on AR signaling**. (**A**), 22Rv1 cells were co-treated with DBF in combination with enzalutamide for 48 h. The viability was measured using the MTT assay and the effect of the drug combination (synergism/additive effect/antagonism) was calculated and visualized using SynergyFinder 2.0 software and a ZIP reference model. Red regions indicate synergism; white—additive effect; green—antagonism. (**B**), Effect of DBF on the expression of several proteins involved in the androgen receptor signaling following 72 h of treatment. Protein expression was accessed using Western blotting. β-actin was used as a loading control.

Hormone therapy is a key component in the treatment of advanced prostate cancer. AR targeting agents (ARTA), e.g., enzalutamide, competitively inhibit the AR resulting in suppression of AR-signaling which in turn leads to inhibition of prostate cancer cell growth and viability [52,55]. Resistance to ARTA is mediated by AR amplification, mutation, or alternative splicing. AR splice variant V7 (AR-V7) lacks a C-terminal androgen binding domain and therefore cannot bind androgens or antiandrogens. An auto-activated AR signaling results in cell proliferation and survival [30]. Along with an AR-full length (AR-FL) expression, 22Rv1 cells are also known to express AR-V7, and therefore are resistant to enzalutamide [29]. Interestingly, DBF is able to resensitize 22Rv1 cells to enzalutamide showing synergistic effects with the ARTA (Figure 7A). In order to explain this phenomenon we examined the effect of DBF on the expressional levels of both AR-FL and AR-V7. Notably, we observed a down-regulation of AR-V7 and other AR splice variants (AR-Vs) in the treated 22Rv1 cells (Figure 7B). Additionally, the expression of AR-FL was also suppressed, suggesting an inhibition of AR signaling (Figure 7B). In line with this, a down-regulation of PSA expression was detected (Figure 7B). Note, PSA is a down-stream target of AR pathway reflecting its activity. Hence, we speculate that DBF is capable of AR-FL/V7-dependent signaling inhibition caused by a down-regulation of both AR-FL and AR-V7 expression. Moreover, due to the suppression of AR-V7 the investigated alkaloid recalls sensitivity of 22Rv1 cells to enzalutamide, making DBF a promising drug candidate for the treatment of castration-resistant PCa.

## 3. Materials and Methods

### 3.1. 3,10-Dibromofascaplysin

The marine alkaloid 3,10-dibromofascaplysin (DBF) was synthesized and purified as previously reported [20]. The purity of the compounds has been confirmed using H^1^ NMR and high-resolution mass spectrometry.

### 3.2. Reagents and Antibodies

CM-H_2_DCFDA was purchased from Molecular probes (Invitrogen, Eugene, OR, USA); MTT (3-(4,5-dimethylthiazol-2-yl)-2,5-diphenyltetrazolium bromide) and propidium iodide (PI)—from Sigma (Taufkirchen, Germany). cOmplete™ *EASY*packs protease inhibitors cocktail and PhosSTOP™ *EASY*packs phosphotase inhibitors cocktail—from Roche (Mannheim, Germany). Anisomycin—from NeoCorp (Weilheim, Germany). N-acetylcystein—from MedChemExpress (Monmouth Junction, NJ, USA). Docetaxel, cabazitaxel, cisplatin and carboplatin—from a Pharmacy of the University Hospital Hamburg-Eppendorf (Hamburg, Germany). RNase—from Carl Roth (Karlsruhe, Germany). Primary and secondary antibodies are listed in Table 2.

### 3.3. Cell Lines and Culture Conditions

The human prostate cancer cell lines DU145, PC-3 and 22Rv1 were purchased from ATCC (Manassas, VA, USA). The docetaxel-resistant DU145-DR and PC3-DR cell lines, generated as described previously [31], were kindly provided by Prof. Z. Culig, Innsbruck Medical University, Austria. The cell lines used had a passage No. ≤ 50, and were continuously kept in culture for a max. of 3 months. All cell lines used were recently authenticated by commercial service (Multiplexion GmbH, Heidelberg, Germany). Cells were cultured as monolayers in a humidified atmosphere with 5% CO_2_ at 37 °C. For cell culture, the RPMI medium supplemented with Glutamax™-I (gibco^®^ Life technologies™, Paisley, UK) containing and 1% penicillin/streptomycin (Invitrogen) and 10% fetal bovine serum (FBS, gibco^®^ Life technologies™) was used. Cells were regularly inspected for stable phenotype and mycoplasma infection.

### 3.4. MTT Assay

Cell viability was evaluated using MTT assay as previously reported [56]. Cells were seeded in 96-well plates (6000 cells/well in 100 μL/well, unless otherwise stated), incubated overnight and the medium was replaced with fresh medium containing the investigated drugs at indicated concentrations. Cells were incubated for the indicated time and MTT reagent was added. After 2 to 4 h of incubation the media was aspirated, the plates were dried (1 h at RT), and 50 μL/well of DMSO were added to each well. The absorbance was measured using Infinite F200PRO reader (TECAN, Männedorf, Switzerland). Cell viability and IC_50_s were calculated using The GraphPad Prism software v. 7.05 was used (GraphPad Prism software Inc., La Jolla, CA, USA)

### 3.5. Kinase Activity Profiling

Kinase activity profiling was performed as previously reported [57,58]. The PamStation^®^12 machine (PamGene International, ’s-Hertogenbosch, The Netherlands) and STK-PamChip^®^ arrays were used for profiling of the affected serine/threonine kinases.

In brief, the cell were lysed using M-PER Mammalian Extraction Buffer (Pierce, Waltham, MA, USA) containing Protease and Phosphatase Inhibitor Cocktails (Pierce). Then, 1 µg of total extracted protein mixture with ATP were applied per each array. The phosphorylation of the specific peptide was detected using primary anti-phospho-Ser/Thr antibodies and secondary polyclonal swine anti-rabbit Immunoglobulin-FITC antibody and CCD camera The signals were further analyzed using the BioNavigator software v. 6.0 (BN6, PamGene International).

### 3.6. Western Blotting

The assay was performed as previously reported [59]. 22Rv1 cells were seeded in Petri dishes (ø 6 cm, 10^6^ cells/well in 5 mL/dish), incubated overnight and treated with the compounds in fresh culture media (5 mL/dish) for indicated time. Then, cells were harvested and the proteins were extracted and separated using PAGE in gradient ready-made gels. The proteins were then transferred onto ø 0.2 µm pore PVDF membrane, followed by blocking and treated with primary and secondary antibodies. The signals were detected using the ECL chemiluminescence system (Thermo Scientific, Rockford, IL, USA). The antibodies used are listed in Table 2.

### 3.7. Determination of Drug Combination Effects

The effects (synergistic versus antagonistic) of MAPK inhibitors (SP600125, SB203580, PD98059, and FR180204), clinically used cytotoxic chemotherapeutics (cisplatin, carboplatin, docetaxel, and cabazitaxel), PARP inhibitor olaparib, or AR inhibitor enzalutamide on the cytotoxic activity of DBF was determined using the Zero interaction potency (ZIP) reference model [43] and the online-based SynergyFinder 2.0 software (https://synergyfinder.fimm.fi, [60]). The experiments were performed as described previously [61]. The 22Rv1 cells (12,000 cells/well of 96-well plate) were co-treated with the defined concentrations of inhibitors and DBF for 48 h in 100 µL/well of 10% FBS/RPMI media. The cytotoxic effects of the individual drugs and its combinations were measured using MTT assay and the data was analyzed using SynergyFinder 2.0 software. Deviations between observed and expected responses with positive (red areas) and negative δ-values (green areas) indicate synergy and antagonism, respectively.

### 3.8. Analysis of Intracellular ROS Level

Intracellular ROS levels were accessed using the flow cytometry technique and CM-H_2_DCFDA staining (Cat. No. C6827, Molecular probes, Invitrogen, Eugene, OR, USA) following the previously reported protocol [62,63]. In brief, 10^5^ of 22Rv1 cells were seeded in 12-well plates and incubated overnight. The media was exchanged with 4 µM CM-H_2_DCFDA solution in pre-warmed PBS (0.5 mL/sample) and the cells were incubated for 30 min in the dark (37 °C, 5% CO_2_). Then the staining solution was replaced with pre-warmed PBS containing investigated compounds at the defined concentrations and the plates were incubated for another 2 h. The cells were harvested and immediately analyzed by flow cytometry.

### 3.9. Analysis of DNA Damage

The effect of the treatment on DNA damage was analyzed using flow cytometry technique and PI staining. The cells were incubated overnight in 6-well plates (0.2 × 10^6^ cells/well in 2 mL/well), then the media was exchanged with fresh drug-containing media and the cells were incubated for another 48 h. After that, the cells were harvested by trypsinization, twice washed with PBS, resuspended in 70% EtOH/H_2_O and incubated overnight at −20 °C. The cells were then pelleted, air-dried, and incubated in 0.2 mL/sample the staining solution containing RNase (0.2 mg/mL) and PI (0.02 mg/mL) in PBS for 30 min in the dark at RT. Then additional 0.2 mL/sample of PBS was added to each sample and the cells were immediately analyzed by FACS Calibur (BD Bioscience, Bedford, MA, USA) instrument. The results were analysed using the BD Bioscience Cell Quest Pro v.5.2.1. software (BD Bioscience, San Jose, CA, USA). The cells containing damaged DNA were identified as sub-G1 population.

### 3.10. Data and Statistical Analysis

For statistical analyses the GraphPad Prism software v. 7.05 was used (GraphPad Prism software Inc., La Jolla, CA, USA). Data are presented as mean ± SD. The unpaired Student’s *t*-test (for comparison of two groups) or one-way ANOVA in combination with Dunnett’s post-hoc test (for comparison of multiple groups) were used to compare the treated groups with control group. All the experiments were performed in triplicates, unless otherwise stated. Statistically significant differences (*) was assumed if *p* < 0.05.

## 4. Conclusions

Here, we investigated the mechanism of action of the marine natural alkaloid 3,10-dibromofascaplysin (DBF), which has been previously identified by us as a promising compound in screening experiments. DBF induced apoptosis in human prostate cancer cells, including hormone- and docetaxel-resistant lines, at low micro- and nanomolar concentrations. No cross-resistance to currently available standard therapies was observed. The kinome analysis followed by the validation and functional experiments identified JNK1/2 as one of the molecular targets of DBF in the cells. Inhibition of JNK1/2 and p38 by specific inhibitors led to the increase of cytotoxic activity of DBF, whereas active ERK1/2 was identified to be important for the cytotoxic activity of the alkaloid. Remarkably, DBF strongly synergized with olaparib due to the induction of ROS production, as well as with other clinically approved platinum and taxane agents. Additionally, the compound inhibited AR-signaling and resensitized AR-V7-positive prostate cancer cells to enzalutamide, presumably due to AR-V7 inhibition. A combination of these properties makes this marine alkaloid a promising drug candidate for the treatment of human castration-resistant PCa.

## Figures and Tables

**Table 1:** Cytotoxicity of DBF in different prostate cancer cells. Cells were incubated with the drug for 72 h. Docetaxel was used as a reference compound.

Cell Line	IC_50_, 72 h	
	DBF [µM]	Docetaxel [nM]
22Rv1	0.29 ± 0.04	0.38 ± 0.08
PC3	0.79 ± 0.17	8.55 ± 3.09
PC3-DR	1.51 ± 0.35	355.8 ± 148.7
DU145	4.19 ± 0.81	1.72 ± 0.19
DU145-DR	1.25 ± 0.27	89.4 ± 13.2

**Table 2:** List of antibodies used.

Antibodies	Clonality	Source	Cat.-No.	Dilution	Manufacturer
anti-AR	pAb	rabbit	sc-816	1:200	Santa Cruz
anti-AR-V7	mAb	rabbit	198394	1:1000	abcam
anti-cleaved Caspase-3	mAb	rabbit	#9664	1:1000	Cell Signaling
anti-ERK1/2	mAb	mouse	#9107	1:2000	Cell Signaling
anti-JNK1/2	mAb	rabbit	#9258	1:1000	Cell Signaling
anti-mouse IgG-HRP		sheep	NXA931	1:10,000	GE Healthcare
anti-p38	mAb	rabbit	#9212	1:1000	Cell Signaling
anti-PARP	pAb	rabbit	#9542	1:1000	Cell Signaling
anti-phospho-ERK1/2	mAb	rabbit	#4377	1:1000	Cell Signaling
anti-phospho-JNK1/2	mAb	rabbit	#4668	1:1000	Cell Signaling
anti-phospho-p38	mAb	rabbit	#4511	1:1000	Cell Signaling
anti-PSA/KLK3	mAb	rabbit	#5365	1:1000	Cell Signaling
anti-rabbit IgG-HRP		goat	#7074	1:5000	Cell Signaling
anti-α-Tubulin	mAb	mouse	T5168	1:5000	Sigma-Aldrich
anti-β-Actin-HRP	pAb	goat	sc-1616	1:10,000	Santa Cruz
